# 

**Published:** 1853-02

**Authors:** 


*The Smelfungus Papers.* — Considerable curiosity is manifested to know the authorship of the Smelfungus papers. The name of the writer will not be disclosed so long as he chooses to remain *incognito.* We will say this much — he does not reside in Buffalo. We hope to be frequently favored with his very clever productions.

---

*Editorial Changes.* — Dr. Geo. Mendenhall has withdrawn from the co-editorship of the Western Lancet, leaving Dr. L. M. Lawson sole editor. The Western Lancet has entered on the fourteenth year of its existence. Dr. Lawson has been connected with it from the commencement.

---

*Cincinnati Retreat for the Insane.* — Dr. Edward Mead, formerly of Chicago, Illinois, has opened a private retreat for the insane, near Cincinnati, Ohio.

---

*Notice.* — On, and after the first day of March next, the address of the editor of this Journal will be *Buffalo, N. Y.*

